# Beneficial Effect of Long-Term Administration of Supplement With Trapa Bispinosa Roxb. and Lutein on Retinal Neurovascular Coupling in Type 2 Diabetic Mice

**DOI:** 10.3389/fphys.2022.788034

**Published:** 2022-02-24

**Authors:** Junya Hanaguri, Harumasa Yokota, Akifumi Kushiyama, Sakura Kushiyama, Masahisa Watanabe, Satoru Yamagami, Taiji Nagaoka

**Affiliations:** ^1^Division of Ophthalmology, Department of Visual Sciences, Nihon University School of Medicine, Tokyo, Japan; ^2^Department of Pharmacotherapy, Meiji Pharmaceutical University, Tokyo, Japan; ^3^Division of Life Science, Department of Nursing, National College of Nursing, Tokyo, Japan

**Keywords:** lutein, retinal blood flow, neurovascular coupling, diabetic model mice, laser speckle flowgraphy

## Abstract

**Purpose:**

We investigated the effect of long-term administration of supplement with trapa bispinosa roxb. extract (TBE) and lutein on the susceptibility of retinal blood flow regulation in type 2 diabetic mice.

**Methods:**

Six-week-old db/db mice were randomly divided into the untreated group (*n* = 6) and the treated group received the supplement with TBE and lutein (*n* = 6). The longitudinal changes in retinal blood flow responses to systemic hyperoxia and a flicker stimulation were evaluated every 2 weeks in diabetes db/db mice from age 8 to 14 weeks. The retinal blood flow was assessed using laser speckle flowgraphy. We also evaluated the expressions of glial fibrillary acid protein (GFAP) and vascular endothelial growth factor (VEGF) by immunofluorescence.

**Results:**

The resting retinal blood flow was steady and comparable between two groups throughout the study. In db/db mice with supplement, both blood flow responses were restored from 8 to 14 weeks of age compared with diabetic mice treated with the placebo. Supplement prevented the activation of GFAP and decreased the expression of VEGF detected by immunofluorescence compared with the diabetic mice treated with placebo.

**Conclusion:**

We found that the long-term administration of supplement with TBE and lutein improved the impaired regulation of retinal blood flow in response to systemic hyperoxia and flicker stimulation, suggesting that these supplements can prevent diabetic retinopathy by improving abnormal neurovascular coupling in type 2 diabetic mice.

## Introduction

Diabetes is a chronic disease that is increasingly prevalent worldwide (Saeedi et al., 2019), and diabetic retinopathy continues as a leading cause of blindness (Bourne et al., 2013). Therefore, the prevention of diabetic microvascular complications is essential. Although there has been ongoing development of invasive therapies, including intravitreal injection, photocoagulation and vitrectomy to treat advanced diabetic retinopathy and/or diabetic macular edema (Gupta et al., 2018; Terasaki et al., 2018), no oral medications or supplements have been developed to prevent advanced diabetic retinopathy. Supplements are a novel candidate for the prevention of diabetic retinopathy. Because some supplements are already approved as food, we can use the supplements as a prevention strategy for patients with no apparent retinopathy.

It was previously reported that glycation and oxidative stress are related to the development of cataract and retinal vessel abnormalities in STZ-induced diabetic rats (Agardh et al., 2000), suggesting that diabetic complications, including cataract and retinopathy, may be related to oxidative stress. Recently, Kinoshita et al. reported that the long-term administration of some commercially available supplements with lutein and trapa bispinosa roxb. extract (TBE), which has a significant antioxidant activity against free radicals, (Malviya et al., 2010) have shown beneficial effects for cataracts in STZ-induced diabetic rats (Kinoshita et al., 2020). In terms of diabetic retinopathy, our recent study revealed that retinal blood flow does not respond to systemic hyperoxia and flicker stimulations in db/db mice with early type 2 diabetes and no apparent changes in resting retinal perfusion (Hanaguri et al., 2021), indicating that early detection of the impaired retinal flow regulation and prompt treatment may protect retinal tissue and prevent or slow the development of irreversible retinopathy. Based on these previous findings, we test the hypothesis that the long-term administration of TBE and lutein supplements may have beneficial effects on the impaired retinal blood flow regulation to flicker stimulation/systemic hyperoxia, and the retinal glial activation and the overexpression of vascular endothelial growth factor (VEGF) in the type 2 diabetic mouse retina.

## Materials and Methods

### Animal Preparation

The Nihon University Ethical Committee approved the animal experiments in the present study, and all experiments were conducted following the tenets of the Association of Research in Vision and Ophthalmology.

One week before the experiment, 5-week-old male C57BL/KsJ-db/db mice (BKS.Cg-Dock7^m^+/+Lepr^db^/J; *n* = 12) and 13-week-old male db/m (non-diabetic congenic littermates, *n* = 5) control mice were purchased from Charles River Laboratories Japan, Inc. (Yokohama, Japan). The mice were housed in a temperature-controlled room with a 12-h dark and light cycle and had free access to food and water. The light level in our animal house was approximately 150–300 lux.

The mice were anesthetized with inhaled 2% isoflurane (Pfizer, Tokyo, Japan) using a constant flow rate of 1.5 L/min for the duration of the experiment. A heated blanket was used to maintain rectal temperature between 37 and 38°C. Blood glucose concentrations were measured from the tail vein (glucose assay kit; Abbott Laboratories, Abbott Park, IL). The pupils were dilated with 0.5% tropicamide (Santen Pharmaceutical Co., Osaka, Japan).

### Chemicals and Systemic Administration Protocol

Trapa bispinosa roxb. extract (67%) was purchased from Hayashikane Sangyo Co., Ltd. (Yamaguchi, Japan), and Lutein (LU) was purchased from Koyo Mercantile Co., Ltd. (Tokyo, Japan). The db/db mice were randomly allocated to either the untreated group (*n* = 6) or the treatment group (*n* = 6). db/db mice in both groups were provided *ad libitum* access to solidified regular food. In the treatment group, the solidified food was mixed with LU (13.3 mg/kg) and TBE (133.3 mg/kg).

### Measurements of Systemic Blood Pressure and Intraocular Pressure

Blood pressure (BP) measurements were made 30 min after induction of anesthesia. The systolic BP (SBP) and diastolic BP (DBP) were measured at the tail with an automatic sphygmomanometer (THC-31, Softron, Tokyo, Japan). The mean arterial BP (MABP) was calculated from the SBP and DBP using the following formula: MABP = DBP + (SBP-DBP)/3.

Intraocular pressure (IOP) was measured with a handheld tonometer (TonolabTV02, ME Technical, Tokyo, Japan), and measurements were made 30 min after induction of anesthesia. Due to the prone position of mice during the experiments, the ocular perfusion pressure (OPP) was calculated with the formula OPP = MABP – IOP (Hanaguri et al., 2020).

### Measurement of Retinal Blood Flow

We used the LSFG-micro system (Softcare Co., Ltd., Fukutsu, Japan) to measure retinal blood flow (Hanaguri et al., 2020). The LSFG has been used for quantitative estimation of ocular [optic nerve head (ONH), choroid, and retina] blood flow in both humans (Sugiyama et al., 2010) and animals (Hanaguri et al., 2020). The LSFG-micro system uses a standard charge-coupled device camera (700 × 480 pixels) equipped with a diode laser (830-nm wavelength) attached to a stereo microscope (SZ61TR, Olympus Corporation, Tokyo, Japan). The principle of LSFG-micro is the same as that of LSFG and is designed for small animals. In brief, the mean blur rate (MBR), representing a relative index of blood velocity, is generated from the blurring of the speckle pattern formed by the backscattered light of the coherent laser by moving blood cells.

To measure retinal blood flow, the mice were positioned on a stand with the right eye facing downward as previously reported. In brief, a cover glass was gently placed on the left cornea with a drop of viscoelastic material. Next, the average MBR of the vessels were analyzed with the LSFG analyzer software program (version 3.2.19.0, Softcare Co., Ltd., Fukutsu, Japan). MBRs were acquired continuously at a rate of 30 frames/s within the area of the O-ring, which was manually placed over the ONH fundus image to mark the margin of the ONH using the LSFG analyzer software program. The MBRs acquired from the ONH area reflect the entire retinal circulation and can be used as an indicator of retinal blood flow (Hanaguri et al., 2020). The MBR outcomes, in response to hyperoxia and flicker light stimulations, were analyzed and expressed as percentage change from the baseline.

### Induction of Hyperoxia

Systemic hyperoxia was induced using the same methodology described in our previous studies (Nagaoka et al., 2016; Song et al., 2016; Zeng et al., 2017; Hanaguri et al., 2020). In brief, 10-min inhalation of 100% oxygen was used to induce hyperoxia. During systemic hyperoxia, we changed the inhalation of isoflurane from room air to pure oxygen through three-way cock. Retinal blood flow measurements were obtained at 1-min intervals for 3 min, and the mean of three consecutive flow measurements was used as the baseline value before the initiation of hyperoxia. During hyperoxia, the retinal blood flow was measured every minute for 20 min (10-min stimulation). Retinal blood flow was also measured every minute during the 10-min recovery following the end of hyperoxia (Hanaguri et al., 2020).

### Flicker Light Stimulation

First, the ambient light was reduced to 1 lux or less, and the mice were dark-adapted for 2 h (Hanaguri et al., 2020). Flicker stimulation was performed with a light intensity of 30 lux, ideal for rod-dominant mouse retina, and a 12 Hz frequency to trigger the maximal retinal blood flow response in mice (Hanaguri et al., 2020). Retinal blood flow was measured with 20 s intervals for 6 min: during the 3-min flicker stimulation and the 3-min recovery. In addition, the mean of three consecutive flow measurements obtained in 1 min (20-s intervals) was used as the baseline value before initiation of flicker light stimulation.

### Experimental Protocols

**Figure 1:**
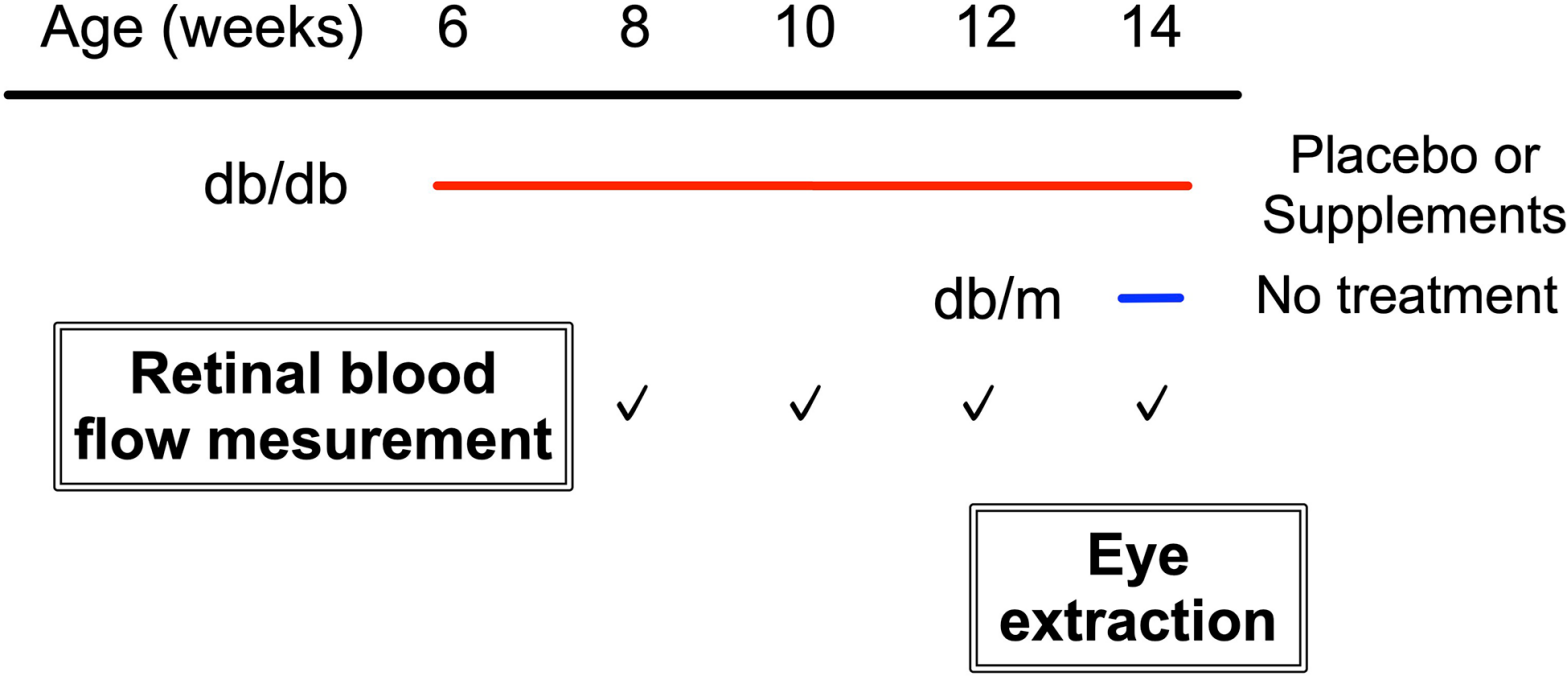
Study protocol.

Figure 1 shows the experimental protocols. Longitudinal assessments of retinal blood flow regulation and neural function were made for each diabetic animal on two consecutive days, every 2 weeks from 8 weeks to 14 weeks of age. In addition, we also performed the same protocol at 14 weeks of age in db/m non-diabetic control mice. The retinal blood flow response to systemic hyperoxia was tested on day one, and the response to flicker light stimulation was evaluated the following day. Of note, systemic BP, IOP, and OPP have been shown to remain unaffected by hyperoxia or flicker light stimulation in mice (Hanaguri et al., 2020).

An independent masked observer (AK) performed all data calculations and analyses.

### Immunohistochemistry

Sternotomy was performed under systemic anesthesia with 3% isoflurane. Normal saline was perfused into the left ventricle to wash out the circulating blood, immediately followed by perfusion of 4% paraformaldehyde (PFA). Eyeballs were enucleated and kept in 4% PFA at 4°C overnight. Then, after a washing twice in phosphate-buffered saline, eyeballs were embedded in Tissue-Tek OCT Compound (Sakura Finetek Japan, Tokyo, Japan) and stored at −80°C until sectioning. Ten-micrometer-thick sections were prepared with a cryostat (HM505, Microm, Walldorf, Germany). Sections were blocked with 10% normal goat serum and stained with glial fibrillary acidic protein (GFAP; ready to use, #Z0034, Dako, Glostrup, Denmark), and VEGF (1:100, #07142 Sigma-Aldrich) overnight at 4°C. Next, they were incubated with secondary donkey anti-rabbit IgG (H + L) Alexa Fluor 488 (dilution, 1:400; Thermo Fisher Scientific) for 2 h at room temperature. The fluorescein intensity was measured using the ImageJ software program (developed by Wayne Rasband, National Institutes of Health, Bethesda, MD; available at http://rsb.info.nih.gov/ij/index.html) after image thresholding and manual exclusion of artifacts.

### Statistical Analysis

Data are expressed as mean ± standard error of the mean. The changes in retinal blood flow were calculated as percentage changes from baseline. The *n* value indicates the number of animals studied. The Kolmogorov–Smirnov test was used to assess the normality of data distribution. Differences among groups and time points were assessed using the one-way or two-way repeated measures ANOVA, as appropriate, followed by Dunnett’s test or Holm–Sidak test, respectively. Prism 9; GraphPad Software, San Diego, California, United States was used to perform all analyses. A value of *p* < 0.05 was considered statistically significant.

## Results

### Longitudinal Assessment of Systemic and Ocular Parameters

**Figure 2:**
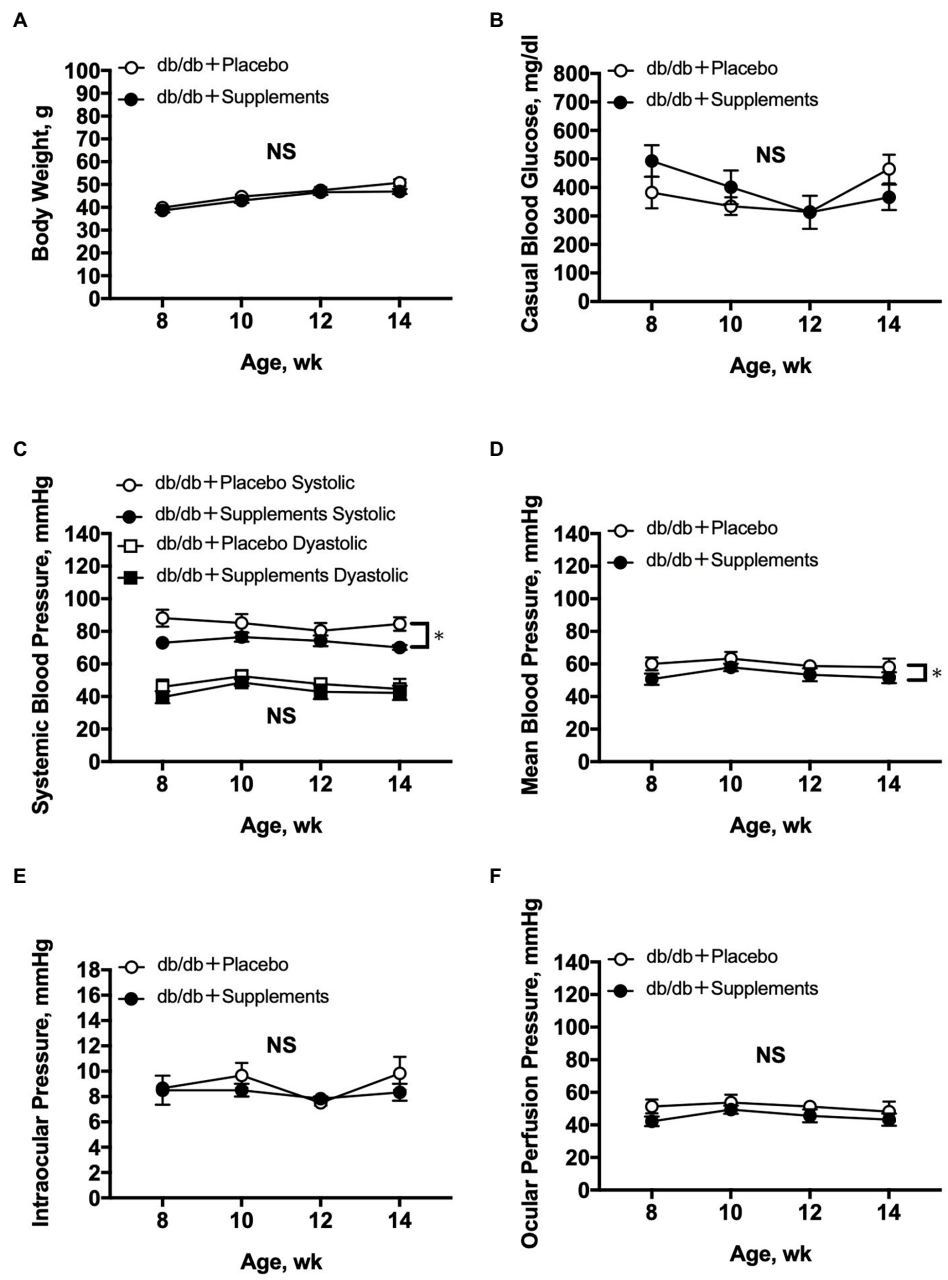
Average systemic and ocular parameters in db/db mice with supplements or placebo from 8 to 14 weeks of age. Significant decreases in systemic blood pressure **(C)** and mean blood pressure **(D)** were observed between db/db with supplements in comparison to placebo-treated db/db mice (*n* = 6; two-way repeated measures ANOVA). In contrast, during the follow-up period no significant differences were observed between the two groups in body weight **(A)**, casual blood glucose **(B)**, diastolic blood pressure **(C)**, intraocular pressure **(E)**, or ocular perfusion pressure **(F)**. NS = not significant between groups. ^*^p < 0.05 between both groups. Data are expressed as the Mean ± SEM.

During the period of experiments, there were no significant differences between treated and untreated mice in terms of body weight, blood glucose level, DBP, IOP, and OPP. In contrast, SBP and MABP were slightly but significantly decreased in db/db mice treated with supplements compared with those in untreated db/db mice (two-way repeated measures ANOVA; Figures 2A-F).

### Longitudinal Assessment of Resting Retinal Blood Flow in Diabetic Mice

**Figure 3:**
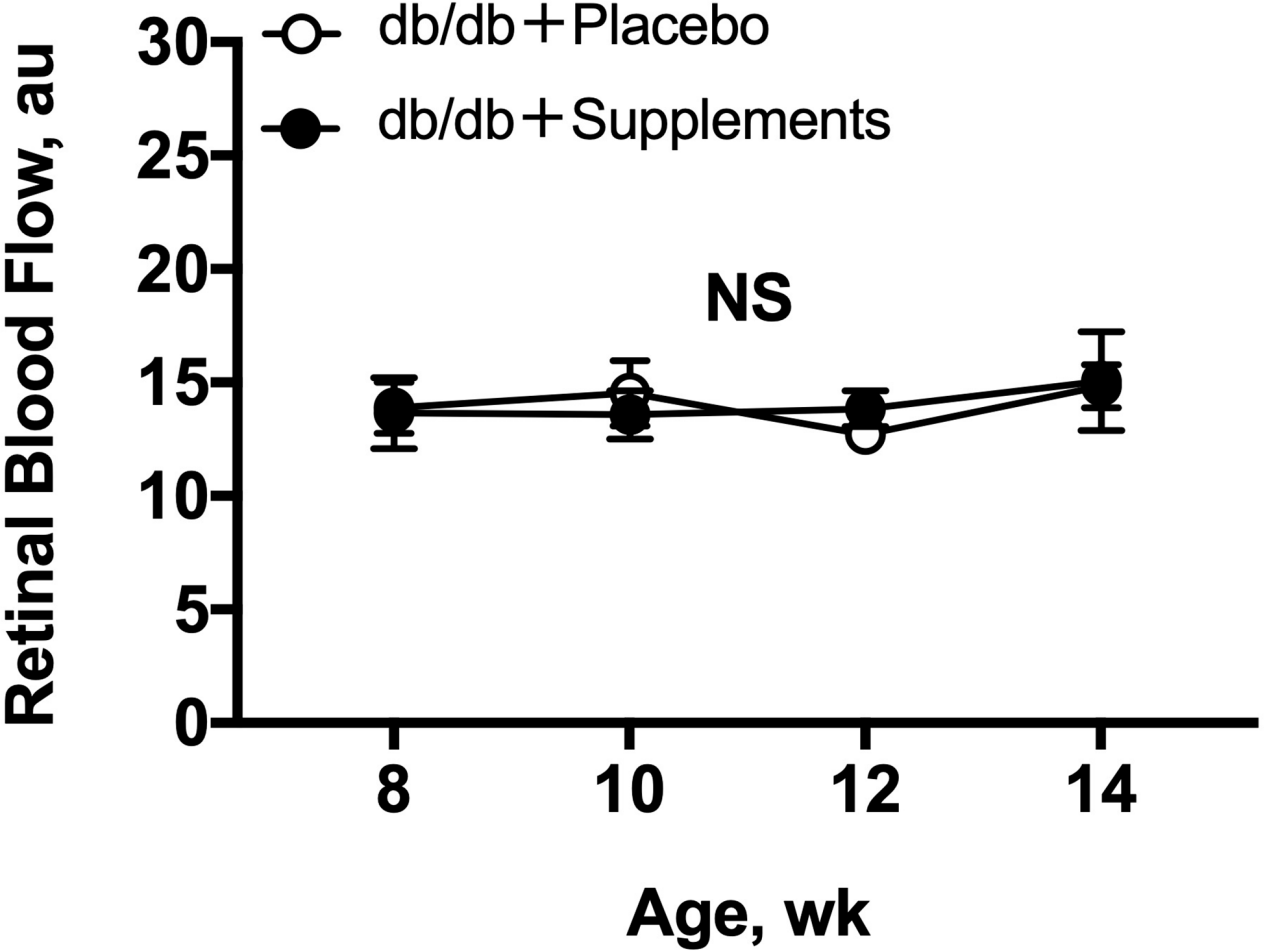
Retinal blood flow in db/db mice with supplements or placebo from 8 to 14 weeks of age. Retinal blood flow (a.u) remained stable in both groups throughout, by one-way repeated measures ANOVA (*p* = 0.88 for db/db with supplements and *p* = 0.50 for db/db with placebo). No differences in resting retinal blood flows were observed (two-way repeated measures ANOVA). NS = not significant between groups and within group.

The resting retinal blood flow is shown in Figure 3. Both untreated db/db and treated db/db showed a steady resting retinal blood flow from 8 to 14 weeks of age with no difference between the groups (two-way repeated measures ANOVA).

### Longitudinal Assessment of Retinal Blood Flow in Response to Systemic Hyperoxia in Diabetic Mice

**Figure 4:**
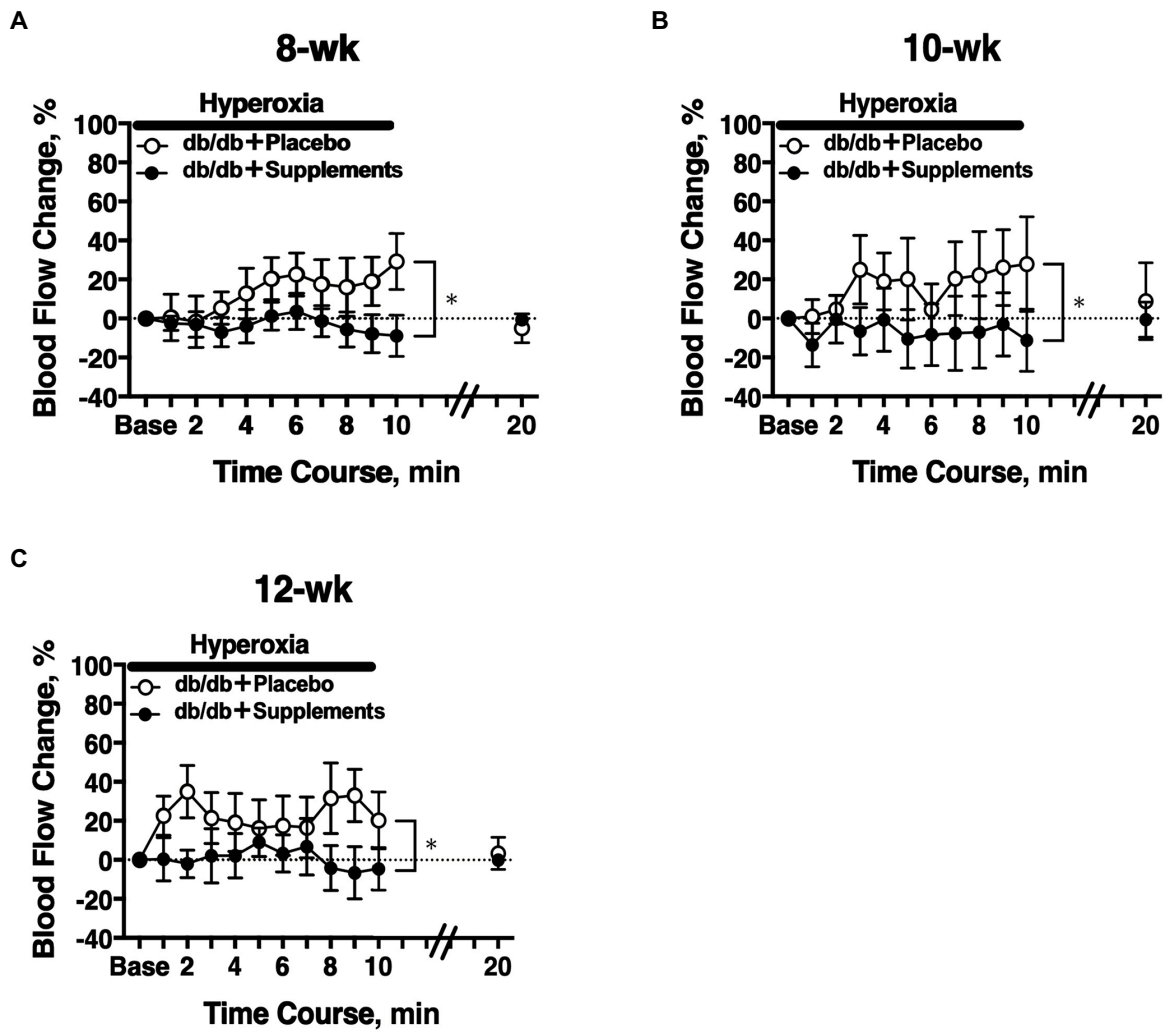
Retinal blood flow response to systemic hyperoxia. Longitudinal assessment of retinal blood flow in db/db mice with placebo or supplements from 8 weeks **(A)**, 10 weeks **(B)**, and 12 weeks **(C)** old. There was a significant difference in the hyperoxia-induced flow response between db/db mice with placebo and supplements for each age studied (two-way repeated measures ANOVA). ^*^p < 0.01 between groups; Solid bar = period of hyperoxia.

In 8 weeks old db/db mice, a significant difference in the hyperoxia-induced change in retinal blood flow was observed between the treatment groups (two-way repeated measures ANOVA; Figure 4A). The significant differences in hyperoxia-induced flow were also observed at 10 weeks and 12 weeks of age (Figures 4B,C).

### Longitudinal Assessment of Retinal Blood Flow in Response to Flicker Stimulation in Diabetic Mice

**Figure 5:**
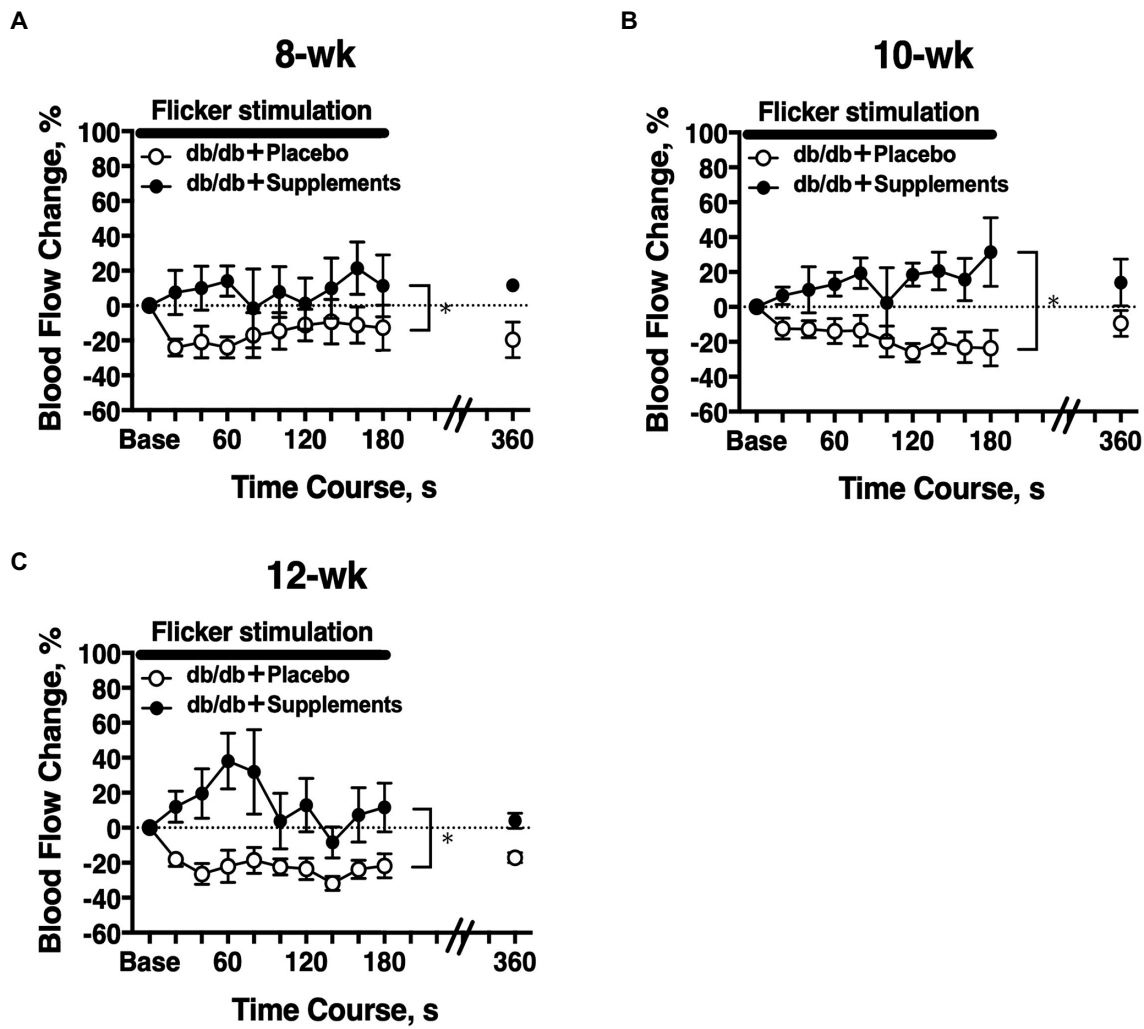
Longitudinal retinal blood flow response to flicker stimulation. Retinal blood flow was longitudinally assessed in db/db mice with supplements (*n* = 6) or placebo (*n* = 6) from 8 weeks **(A)**, 10 weeks **(B)**, and 12 weeks **(C)** old. There was a significant difference in the flicker-induced flow response between db/db mice with placebo and supplements for each age studied (two-way repeated measures ANOVA). ^*^p < 0.01 between groups; Solid bar = period of flicker stimulation.

The temporal change in retinal blood flow in response to a 3-min flicker light stimulation was evaluated on experiment day 2. In 8-week-old db/db mice, there was a significant difference in the flicker-induced change in retinal blood flow between untreated and treated mice (two-way repeated measures ANOVA; Figure 5A). The significant differences in flicker-induced flow changes were also observed at 10 weeks and 12 weeks of age (Figures 5B,C).

### Comparison of the Change in RBF in Response to Hyperoxia and Flicker Stimulation at 14 Weeks of Age in Diabetic Mice With db/m Non-diabetic Control Mice

The same protocol of systemic hyperoxia and flicker stimulation was repeated at 14 weeks.

**Figure 6:**
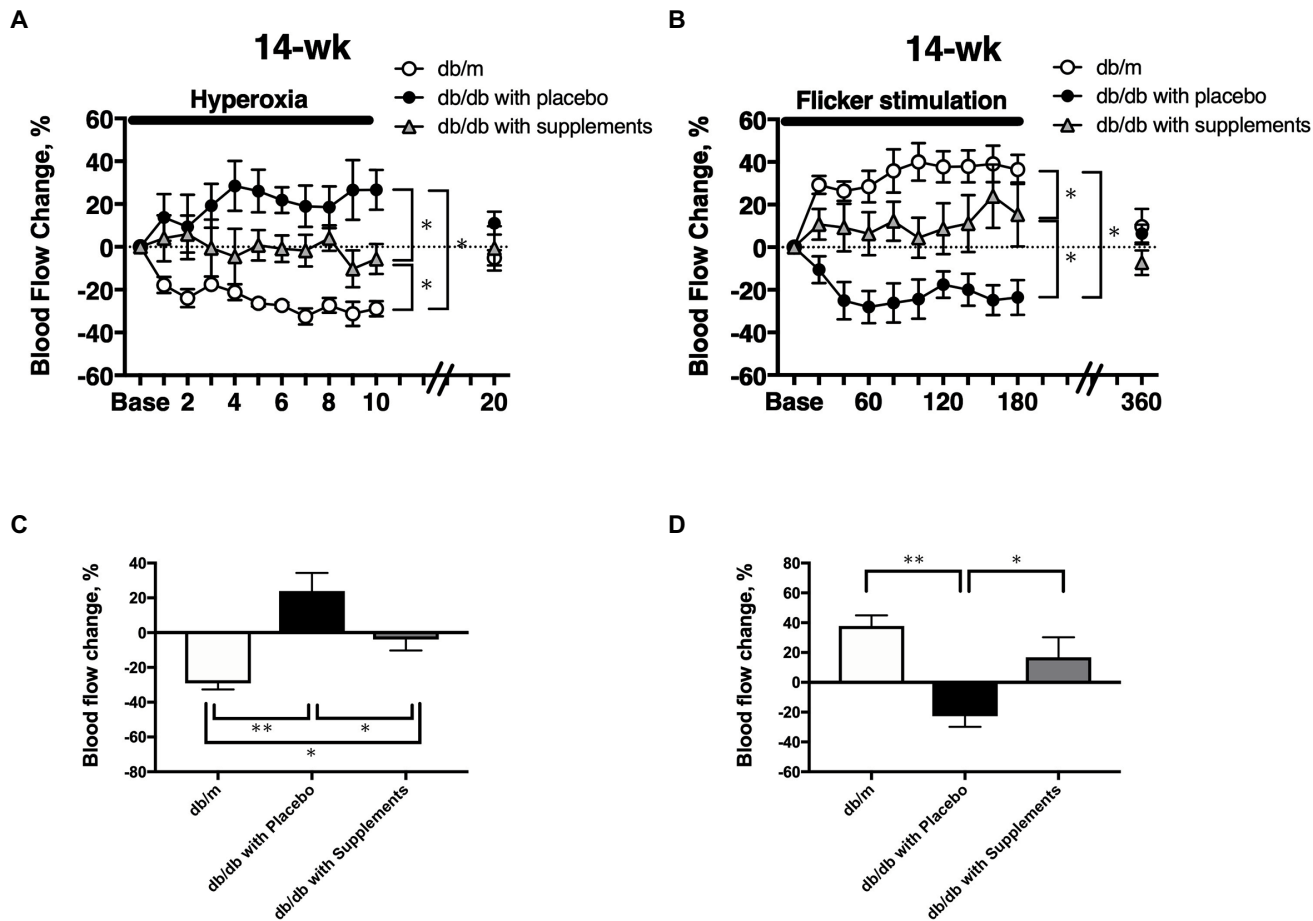
The time course of the changes in retinal blood flow in response to systemic hyperoxia **(A)** and flicker stimulation **(B)** at 14 weeks of age among the three groups. The late-phase maximum changes in retinal blood flow from baseline in response to hyperoxia **(C)** and flicker stimulation **(D)** in 14-week-old mice of db/m (*n* = 5) as non-diabetic control and db/db with placebo (*n* = 6) and supplements (*n* = 6). ^*^p < 0.05, ^**^p < 0.01 compared with db/m by one-way ANOVA followed by Holm-Sidak test.

At 14 weeks of age, there were significant differences among the three groups in the changes in retinal blood flow in response to systemic hyperoxia (Figure 6A) and flicker stimulation (Figure 6B). In non-diabetic control db/m mice, the retinal blood flow decreased and increased from the baseline values (one-way repeated measures ANOVA: *p* < 0.01 and *p* < 0.05). in response to systemic hyperoxia and flicker stimulation, respectively. In untreated diabetic mice, the retinal blood flow showed the opposite responses to systemic hyperoxia and flicker stimulation. The retinal blood flow tended to increase and decrease in response to systemic hyperoxia and flicker stimulation; however, these changes from the baseline values were not statistically significant (one-way repeated measures ANOVA: *p* = 0.44 and *p* = 0.14). In the supplement group, these changes seemed to be improved, but were approximately half that of the db/m non-diabetic control group. To compare the maximum responses in the late phase of each challenge, we calculated the late-phase maximum responses as the averaged changes in retinal blood flow values that were obtained at the last three time points during each challenge (from 140 to 180 s during flicker stimulation and from 8 to 10 min during systemic hyperoxia). The changes of retinal blood flow in response to systemic hyperoxia and flicker stimulation that were observed in non-diabetic db/m mice were not present in the non-treated db/db mice; however, in the supplement group these changes were significantly improved in comparison to the untreated diabetic mouse group; although they did not recover to the same levels as the db/m control group (Figure 6C).

### Beneficial Effect of Systemic Administration of Supplements on GFAP and VEGF Expression

**Figure 7:**
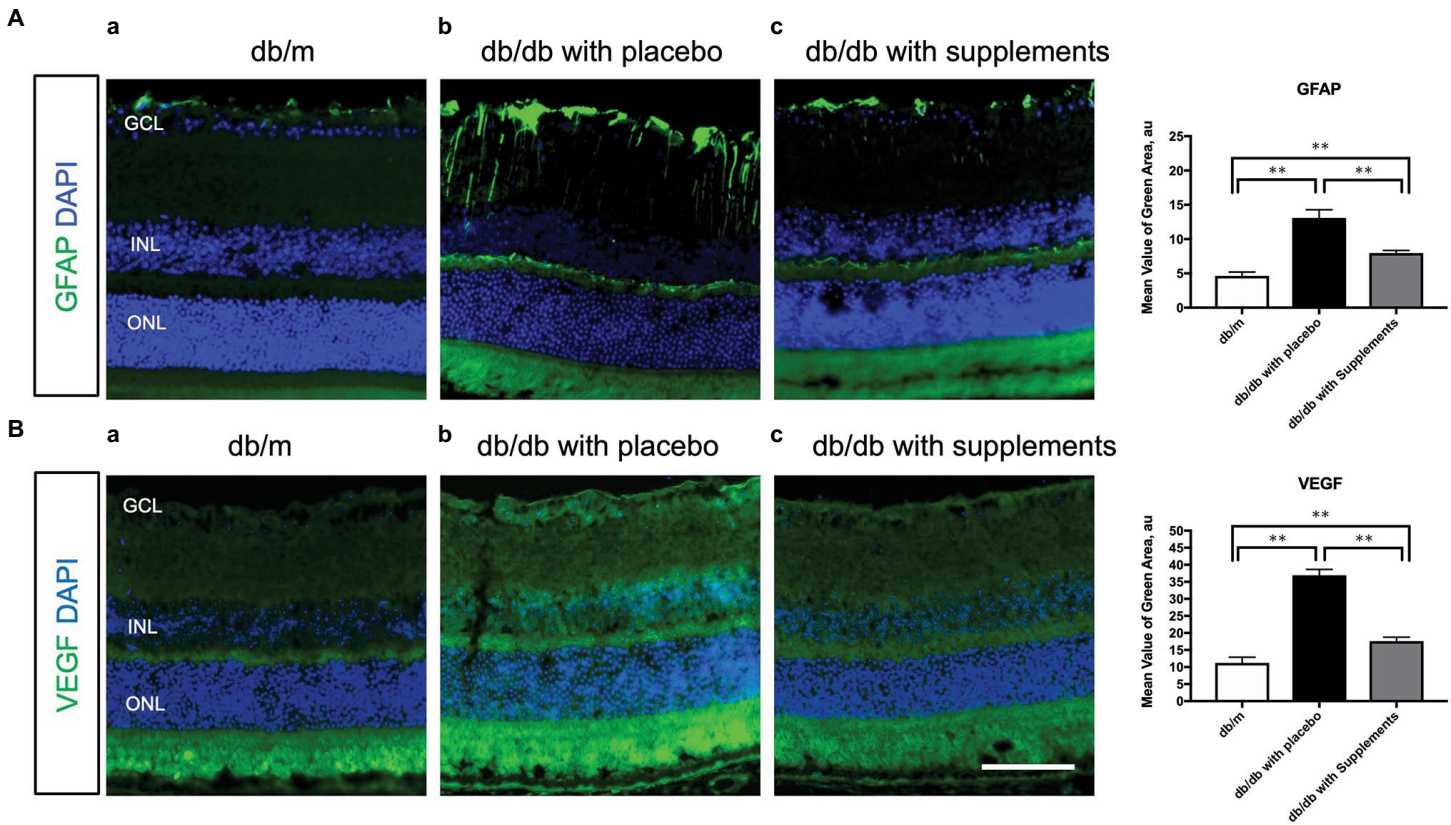
Effect of supplements on the glial activation **(A)** and the VEGF production **(B)**. **(A)** Comparison of GFAP immunofluorescence (green) between representative samples from in db/m (a) and db/db mice with placebo or supplements at 14 weeks. In the diabetic retina with placebo, Müller cells’ endfeet show abundant GFAP immunofluorescence and the radial processes stain intensely throughout both the inner and outer retina. In the diabetic retina with supplements, the GFAP expression of Müller cells endfeet was reduced and was comparable to that in db/m control mice. **(B)** Representative images of the VEGF expression in each group at 14 weeks of age. The VEGF expression was increased in the whole layers of the retina in db/db mice treated with placebo. The VEGF expression pattern of db/db mice treated with supplements was almost comparable to that of db/m mice. ^*^p < 0.05, ^**^p < 0.01 by one way ANOVA followed by Holm-Sidak test. Nuclei were counterstained with DAPI (blue). ONL, outer nuclear layer; INL, inner nuclear layer; GCL, ganglion cell layer. VEGF, vascular endothelial growth factor. Scale bar = 100 μm.

To clarify the beneficial effect of supplements on the pathogenesis of diabetic retinopathy, we performed immunohistochemical analysis to investigate whether long-term systemic administration of supplements ameliorates the glial activation and hyperpermeability in the retina of the type 2 diabetic murine model. As shown in Figure 7, supplements decreased the expression of GFAP (Figure 7A) and VEGF (Figure 7B) in the retina of db/db mice treated with supplements. The fluorescein intensities of GFAP and VEGF in the retina were significantly increased in untreated db/db mice in comparison to non-diabetic db/m mice and db/db mice treated with supplements (Figure 7).

## Discussion

The present study’s findings suggest that the long-term administration of supplement with TBE and lutein improve retinal blood flow dysregulation in response to systemic hyperoxia and flicker stimulation and prevent the Müller glial dysfunction and VEGF expression.

It is well known that the metabolic abnormalities of diabetes cause mitochondrial superoxide overproduction in endothelial cells, which increases the formation of advanced glycation end (AGE) products (Giacco and Brownlee, 2010). In addition, Curtis et al. reported that diabetes was associated with the upregulation of oxidative stress and the induction of GFAP production in Müller glial cells in STZ-induced diabetic rats (Curtis et al., 2011). The aberrant overexpression of GFAP, which is induced after retinal damage (Lewis and Fisher, 2003), was observed in Müller cells in the diabetic retina (Mizutani et al., 1998; Cheung et al., 2005). In addition, they also found that AGE formulation was linked to the Müller glial cell dysfunction (Curtis et al., 2011). Because we have previously found that retinal glial cells play important roles in the change in retinal blood flow in response to systemic hyperoxia (Song et al., 2016) and flicker stimulation (Song et al., 2015), the impaired blood flow response to hyperoxia and flicker observed in diabetic mice may be caused by the Müller glial cell dysfunction induced by the accumulation of AGE. Trapa bispinosa roxb. is an aquatic floating herb and contains large amounts of non-nutritional antioxidants, including flavonoids (Adkar et al., 2014). *In vitro* study revealed that trapa bispinosa roxb. has significant antioxidant activity against free radicals (Malviya et al., 2010). Recently, Kinoshita et al. reported that the systemic administration of TBE (100 mg/kg) and lutein (10 mg/kg) for 29 days inhibited the increase in AGEs products in the retina of STZ-induced diabetic rats (Kinoshita et al., 2020). Although the diabetic animal model differed from the type used in our study, the doses of TBE and lutein were almost identical to those in our study. Although we could not measure the concentration of AGEs in the serum and retina in the current study, the improvement of retinal vascular dysregulation, Müller glial cell dysfunction, and the overexpression of VEGF observed in untreated db/db mice may be associated with the decreased AGEs and oxidative stress in the serum and/or retina induced by supplementation with TBE and lutein.

Notably, no significant change in blood glucose was observed in either the untreated or supplement-treated db/db mice. These results are comparable with the previous study using STZ-induced diabetic rats (Kinoshita et al., 2020). The long-term administration of the supplement on systemic and ocular parameters had little influence on our results. However, systolic BP and mean BP were slightly, but significantly, decreased in treated db/db mice compared with untreated db/db mice. There was no previous study to examine the effect of supplement with TBE and lutein on systemic blood pressure. Despite the decrease in systemic blood pressure by oral supplementation with TBE and lutein, the calculated OPP did not significantly change from 8 to 14 weeks of age (Figure 2). This difference suggests that the effect of the supplements on systemic blood pressure seems to be small with little or no influence on retinal blood flow responses.

The long-term application of TBE and lutein for 8 weeks restore the retinal blood flow regulation in response to systemic hyperoxia (Figures 4, 6A). Told et al. reported that the supplement including lutein for 14 days can normalize retinal blood flow response during 100% O_2_ breathing under LPS administration in humans (Told et al., 2014). Although there were differences in the contents of the supplements other than lutein and disease model (endotoxin-induced oxidative stress model vs. type 2 diabetes mellitus) between their human study and our animal study, our finding about the restoration of blood flow response to hyperoxia by systemic administration of lutein complex is comparable with their result.

We have previously reported that retinal glial cells play important roles in retinal blood flow regulation in systemic hyperoxia and flicker stimulation (Song et al., 2015), *via* the production of endothelin-1 (ET-1; Song et al., 2016) and nitric oxide (NO; Yoshioka et al., 2015) in anesthetized cats. It was also reported that NO-dependent dilation of porcine retinal arterioles was reduced in STZ-induced diabetic pigs, which indicated the endothelial dysfunction in the retinal arterioles (Hein et al., 2012). Although we did not measure the level of ET-1 and NO in the diabetic retina, the improvement of Müller glial dysfunction by the long-term treatment of supplements may be associated with the improvement of retinal endothelial function, resulting in the restoration of retinal blood flow dysregulation in type 2 diabetic mice.

Riva et al. reported an increase in retinal blood flow when neuronal retina are stimulated by flickering light (Riva et al., 1991). This hyperemic response is thought to be mediated neurovascular coupling in the retina, a linkage of neural activity and metabolism for blood flow regulation (Newman, 2013). Pathological neovascularization and retinopathy in diabetes blunts this retinal blood flow response to systemic hyperoxia (Grunwald et al., 1989) and flicker light (Mandecka et al., 2007). We have found that the increases in RBF in response to flicker stimulation were regulated partly by neuronal NOS (Yoshioka et al., 2015) and retinal glial cells in anesthetized cats (Song et al., 2015). We also found a gradual decrease with age in the flicker stimulation-induced hyperemia. Another finding of note was the confirmation that the glial activation detected by GFAP in the retina was induced in the untreated db/db mice; however, glial activation was reduced with the TBE and lutein supplement (Figure 7). Considering all the above, the return of the retinal blood flow response to flicker stimulation following administration of TBE and lutein supplements (Figure 5) suggests that these supplements can restore the retina’s neurovascular coupling by the inhibition of the Müller cell activation induced by diabetes.

We have previously confirmed that the retinal blood flow response to systemic hyperoxia and flicker stimulation remains stable in db/m non-diabetic control mice from 8 to 20 weeks of age (Hanaguri et al., 2021). Due to the animal welfare criteria in our institution, we performed only one retinal blood flow at 14 weeks of age in db/m mice. We found that maximum responses of retinal blood flow in response to hyperoxia and flicker stimulation in db/db mice with supplements were not fully recovered as the same as those in non-diabetic db/m mice at 14 weeks of age (Figure 6), suggesting that the supplement can almost restore the normal responses of the retinal blood flow to systemic hyperoxia and flicker stimulation. Further study is warranted to confirm the effect of supplement on the prevention of diabetic retinopathy in diabetic animal model which expressed diabetic retinopathy or human diabetic patients.

Müller cell gliosis, indicated by GFAP expression, is the most sensitive, non-specific response to retinal injury (Lewis and Fisher, 2003) and is involved in retinal cell death in db/db mice (Bogdanov et al., 2014). In the present study, we also found that that long-term supplement decreased the GFAP expression in db/db mice at 14 weeks of age (Figure 7). These findings indicate that supplement restored retinal blood flow dysregulation in response to the systemic hyperoxia and flicker stimulation in diabetic mice. Sasaki et al. examined the neuroprotective effects of lutein against retinal neural damage caused by inflammation in endotoxin-induced uveitis model (Sasaki et al., 2009). They found that pathologic change of Müller glial cells, represented by GFAP expression, was prevented by lutein. Although there is no study using a diabetic model to examine the effect of lutein on the GFAP expression, (Bogdanov et al., 2014) lutein and/or TBE may have a beneficial effect on the glial activation in the retina in type 2 diabetic mice.

In db/db mice, we observed that was also increased and the supplement reduced the overexpression of VEGF in db/db mice at 14 weeks of age (Figure 7). Xiong et al. reported an increase in VEGF in retinas of db/db mice accompanied by increased retinal vascular leakage (Xiong et al., 2017). These results are comparable with our current findings. Recently, it has been reported that lutein administration also reduced the upregulation of VEGF in the diabetic Akita mouse retina (Wang et al., 2020), suggesting that lutein may help to reduce pathologic VEGF levels by suppressing augmented oxidative stress and inflammatory activities. Although there was no study to examine the effect of TBE *per se* on the expression of VEGF, our result showed that supplements with TBE and lutein can reduce the VEGF expression, which is expected to be a therapeutic potential for not only the diabetic retinopathy but also diabetic macular edema.

Although some previous studies have reported that retinal neuropathy can occur either before or at the same time as vasculopathy (Bui et al., 2003; Yee et al., 2010), especially in humans (Adams and Bearse, 2012), we previously found that the impaired retinal blood flow regulation in response to flicker stimulation and systemic hyperoxia occurred from 8 weeks of age whereas the retinal neuronal abnormalities detected by electroretinogram (ERG) were observed from 14 weeks of age in db/db mice, (Hanaguri et al., 2021) suggesting that the abnormal blood flow regulation and the impaired neurovascular coupling in the retina may precede the neuronal change in type 2 diabetic mice. Therefore, we hypothesize that long-term supplementation of lutein and TBE may have a more beneficial effect on the vascular function in comparison to the neuronal function *via* the reduction of AGE accumulation and oxidative stress in the diabetic retina. Further studies using ERG recording and a longer observation period are warranted to test our hypothesis.

## Limitations

The following limitations should be considered when interpreting the results. First, we used only the supplements with TBE and lutein, so it is difficult to confirm whether lutein or TBE or both may have a beneficial effect on the retinal blood flow dysregulation. Second, there were few studies to examine the molecular mechanism of effect of TBE on diabetic retinopathy whereas TBE has been used as the supplement of eye disease in Japan. Although previous review paper reported that TBE may reduce oxidative stress and decrease the production of AGEs (Adkar et al., 2014), we could not examine the detailed mechanism of the effect of TBE on the protective effect on the retinal blood flow dysregulation in db/db mice. Further clinical study is needed to confirm the beneficial potential of TBE *per se* on diabetic retinopathy. Third, we did not examine the effect of TBE and lutein on the inhibition of the progress of diabetic retinopathy because we could not observe any apparent histological changes in the retina in db/db mice at 14 weeks of age. Further study is warranted to confirm this effect using animal models with apparent abnormalities induced by diabetes.

Fourth, since the supplements were only mixed into the food, the amount that the animals actually ingested may be uncertain. Finally, we could not measure arterial oxygen saturation during systemic hyperoxia in mice because it was not possible to obtain a sufficient amount of blood from living mice.

## Conclusion

The current results suggest the long-term administration of supplements with trapa bispinosa roxb. and lutein can improve retinal blood flow dysregulation in response to systemic hyperoxia and flicker stimulation and prevent the glial dysfunction and VEGF expression in type 2 diabetic mice. With substantial efficacy, trapa bispinosa roxb. and lutein may serve as a reliable and promising agent for the early intervention of diabetic retinopathy and delay its progression when traditional invasive treatments, such as anti-VEGF injection, laser photocoagulation, and intravitreal steroid therapy, are required.

## Data Availability Statement

The raw data supporting the conclusions of this article will be made available by the authors, without undue reservation.

## Ethics Statement

The animal study was reviewed and approved by the Nihon University Ethical Committee.

## Author Contributions

TN: conceptualization. TN, HY, and AK: methodology, writing—review, and editing. AK: formal analysis. JH, HY, AK, SK, and MW: investigation. SY and TN: resources and funding acquisition. TN and JH: writing—original draft preparation. All authors contributed to the article and approved the submitted version.

## Funding

This research was supported by a Grant-in-Aid for Scientific Research (C) 26861430 from the Ministry of Education, Science, and Culture, Tokyo, Japan, a grant from the Uehara Memorial Foundation, and a research fund from Santen Pharmaceutical Co., Ltd. (to TN).

## Conflict of Interest

The authors declare that the research was conducted in the absence of any commercial or financial relationships that could be construed as a potential conflict of interest.

## Publisher’s Note

All claims expressed in this article are solely those of the authors and do not necessarily represent those of their affiliated organizations, or those of the publisher, the editors and the reviewers. Any product that may be evaluated in this article, or claim that may be made by its manufacturer, is not guaranteed or endorsed by the publisher.
